# A cost-optimized 5-protein panel revolutionizes systemic lupus erythematosus diagnosis

**DOI:** 10.1371/journal.pcbi.1014513

**Published:** 2026-07-23

**Authors:** Wenhua Lv, Zhenwei Shang, Chen Sun, Yuping Zou, Siyu Wei, Haiyan Chen, Junxian Tao, Hongsheng Tian, Yu Dong, Chen Zhang, Mingming Zhang, Hongchao Lv, Yongshuai Jiang

**Affiliations:** College of Bioinformatics Science and Technology, Harbin Medical University, Harbin, China; Guangxi University, CHINA

## Abstract

Early diagnosis of systemic lupus erythematosus (SLE) is hindered by a lack of reliable biomarkers. This study sought to identify and evaluate diagnostic plasma protein biomarkers for SLE. We analyzed plasma protein profiles, polygenic risk scores (PRS), and clinical data from 544 SLE cases and 48,036 controls in the UK Biobank. Using LASSO regression, we identified 35 high-confidence SLE-associated proteins and derived a protein risk score (ProtRS). The ProtRS model achieved exceptional diagnostic performance (AUC = 0.91), significantly outperforming models based on PRS or clinical factors alone. Notably, a cost-optimized 5-protein panel (TRIM21, SOD2, KLK3, IL15, ADIPOQ) retained high accuracy (AUC = 0.82) while reducing costs by ~87%. ProtRS also demonstrated the highest population attributable fraction (96.34%), underscoring its dominant contribution to SLE burden. This study establishes a protein-driven framework for early SLE detection, offering tiered diagnostic solutions to balance accuracy and cost. The findings underscore the translational potential of protein biomarkers in bridging theoretical research and clinical practice.

## Author summary

Systemic lupus erythematosus (SLE) is a severe autoimmune disease that primarily affects women, yet early diagnosis remains challenging due to a lack of reliable biomarkers. Current diagnostic approaches rely on clinical symptoms and autoantibody tests that suffer from poor sensitivity or specificity. In this study, we leveraged plasma protein data from over 48,000 individuals in the UK Biobank to identify protein biomarkers for SLE. Using a balanced case-control sampling strategy combined with machine learning, we identified 35 proteins that accurately distinguish SLE patients from healthy controls. Furthermore, we developed a cost-optimized panel of just five proteins (TRIM21, SOD2, KLK3, IL15, and ADIPOQ) that maintains high diagnostic accuracy while reducing testing costs by approximately 87%. This 5-protein panel can be readily implemented in routine clinical laboratories using existing immunoassay platforms, offering a practical and affordable solution for early SLE detection. Our findings provide a protein-driven framework that bridges computational discovery and clinical application, potentially improving outcomes for patients with this debilitating disease.

## Introduction

Systemic lupus erythematosus (SLE) is a chronic autoimmune disorder characterized by significant gender disparity, with women exhibiting markedly higher incidence rates than men. The disease manifests with remarkable clinical heterogeneity, ranging from mild cutaneous manifestations to severe multi-organ involvement and life-threatening complications [1, 2]. Notably, it represents a leading cause of mortality among young women, as evidenced by a comprehensive meta-analysis encompassing over 26,000 female SLE patients. For female SLE patients, it revealed an all-cause mortality ratio of 2.6-fold, a 2-fold increase for cardiovascular disease mortality and nearly 5-fold increase for infection and renal disease-related mortality when compared with the general population [3]. While the global prevalence and incidence of SLE demonstrate considerable geographical variation, it consistently imposes high disease burden across populations and substantial economic costs associated with long-term management [4, 5]. These compelling data underscore the critical importance of early diagnostic strategies, timely therapeutic interventions and comprehensive risk factor management.

Current diagnostic approaches for SLE, which rely on clinical manifestations (e.g., malar rash, photosensitivity, oral ulcers, arthritis) and routine laboratory tests (e.g., anemia, leukopenia, proteinuria), pose significant challenges for early-stage detection. While established serological biomarkers, particularly autoantibodies such as antinuclear antibodies (ANA) and anti-Smith (anti-Sm) antibodies remain cornerstones of diagnosis, their limitations in balancing sensitivity and specificity hinder clinical utility. For instance, ANA (1:80) demonstrates >95% sensitivity but poor specificity, whereas anti-Sm antibodies exhibit >90% specificity yet poor sensitivity [6, 7].

Recent advances in SLE risk stratification have predominantly focused on gene expression profiling [8–10], yet large-scale protein biomarker discovery remains under-explored, largely due to historical limitations in high-quality protein datasets. The emergence of the UK Biobank (UKB) [11], with its extensive plasma protein data, now provides an unprecedented opportunity to address this gap. Protein biomarkers hold unique advantages over transcriptomic indicators in SLE management: (1) Functional Relevance: As direct executors of biological processes, plasma proteins dynamically reflect pathological changes in inflammation, immune dysregulation, and metabolic pathways [12]. (2) Early Detection Potential: protein abnormalities often precede clinical symptoms, as exemplified by glial fibrillary acidic protein (GFAP) elevations a decade before Alzheimer’s disease onset [13, 14]. (3) Dynamic Monitoring: Protein levels fluctuate with disease activity and therapeutic responses, enabling real-time tracking [15], which is a critical feature for managing SLE’s relapsing-remitting nature.

This study leverages UKB’s protein resources to systematically identify robust SLE biomarkers, aiming to overcome the sensitivity-specificity trade-off of conventional autoantibodies. Using a balanced case-control sampling (BCCS) approach to address case-control imbalance, we aim to systematically screen and validate stable SLE-associated protein signatures through iterative resampling (n = 1,000 repetitions), calculate protein risk scores (ProtRS) by integrating biomarker weights, and then develop an SLE prediction model with performance evaluation. A key innovation for this research lies in constructing a cost-optimized prediction model that maintains high diagnostic accuracy (AUC > 0.80) while minimizing patient expenses by prioritizing a parsimonious biomarker panel (e.g., top 5 proteins). This approach bridges the gap between basic research and clinical utility, offering a tiered testing options (5/10/20 biomarkers) adaptable to healthcare resource settings and an actionable insights for early SLE detection and precision prevention strategies. By combining robust statistical methods with cost-effectiveness considerations, this study provides a framework for biomarker-driven SLE risk assessment and scalable diagnostic solutions in real-world practice.

## Results

### Population characteristics overview of SLE and control individuals

The number of SLE patients and control individuals included in this study was 544 and 48,306, respectively. There were 25,618 females and 22,418 males in the control group, respectively, while the number of female patients was 459 in the SLE group, accounting for 84.4%. The age of SLE patients ranged from 9 to 84 years, with a mean age of 61 years and a standard deviation of 12, where no significant difference in the mean age of females and males was observed. The detailed information for population characteristics can be found in Table 1.

**Table 1:** Characteristics summary for study cohort.

Characteristics	SLE	Control
Female, n (%)	459 (84.4%)	25618 (53.3%)
Age (mean ± sd)	61.2 ± 12.2	64.9 ± 9.5
PM25 (mean ± sd)	10.15 ± 1.09 (n = 507)	10.01 ± 1.07 (n = 44112)
Smoker, n(%)	263 (48.3%)	21558 (44.9%)
Sleep duration, n (%)		
≤6 hours	169 (31.5%)	11664 (24.5%)
7-8 hours	290 (54.1%)	32119 (67.4%)
> 9 hours	77 (14.4%)	3861 (8.1%)
Insomnia, n (%)		
1 (never or rarely)	72/542 (13.3%)	11668/47930 (24.3%)
2 (sometimes)	264/542 (48.7%)	22839/47930 (47.7%)
3 (usually)	206/542 (38%)	13423/47930 (28%)

### Protein risk score well distinguishes SLE from control samples

**Fig 1:**
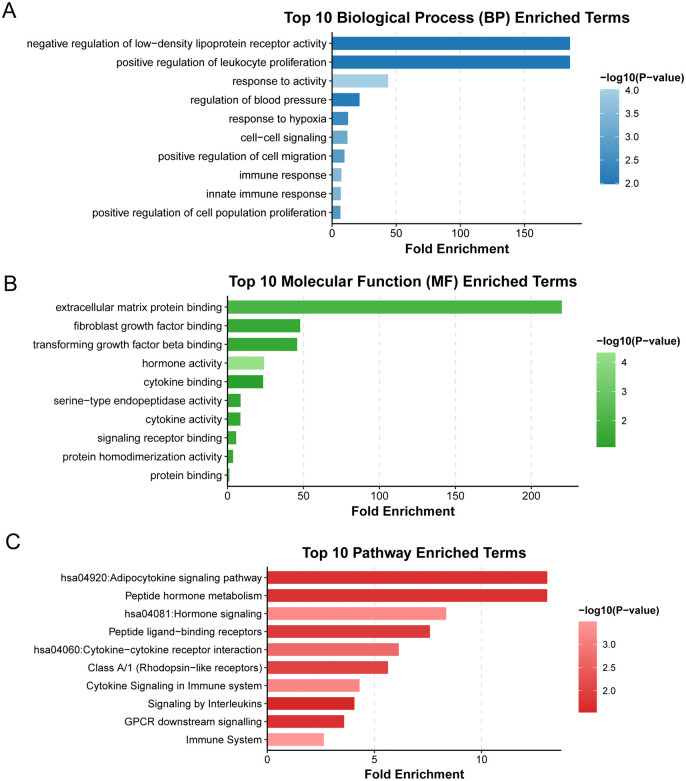
Function annotation for 35 SLE-associated risk proteins. **(A)** Top 10 biological process (BP) enrichment terms. **(B)** Top 10 molecular function (BP) enrichment terms. **(C)** Top 10 pathways enrichment terms.

A total of 35 protein markers that were screened out in at least 500 random LASSO regressions were obtained, of which 18 proteins were positively correlated with SLE and the remaining 17 proteins were negatively correlated with SLE. The distribution of regression coefficients for these proteins is shown in the S1 Fig and S1 Table. These proteins are mainly involved in biological processes related to immune response and inflammatory response, exert molecular functions including chemokine activity, cytokine activity, and hormone activity, and play an important role in cytokine receptor interaction and human immunodeficiency virus 1 infection-related pathways (Fig 1, S2 Table). The top five proteins with the highest number of repetitions in 1000 BCCS were TRIM21, SOD2, KLK3, IL15 and ADIPOQ, all of which achieved over 95% selection frequency. High reproducibility of multiple random samplings and functional annotations indicate that these 35 protein markers are indeed closely related to SLE, which lays the foundation for subsequent prediction model construction.

**Fig 2:**
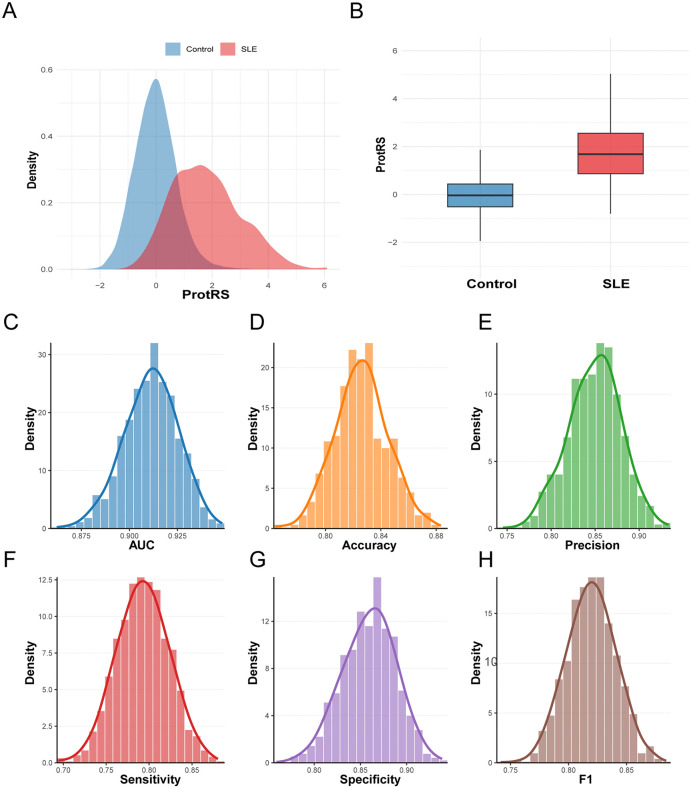
The performance of ProtRS in distinguishing SLE patients from controls. **(A)** Density plot of ProtRS for both SLE and control groups. **(B)** A box plot comparing ProtRS between two groups. **(C-H)** Distribution of metrics evaluating the performance for 35-protein model. It refers to the results of AUC **(C)**, Accuracy **(D)**, Precision **(E)**, Sensitivity **(F)**, Specificity **(G)**, and F1 **(H)**, respectively.

Patients with SLE had significantly higher protein risk scores than those in the control group, which was calculated by the 35 SLE risk proteins (Fig 2A, 2B). After adjusting for age and gender, the full ProtRS-based model were able to well distinguish SLE patients from control samples. Specifically, the average OR for ProtRS reached 9.20 (95% UI: 6.81 - 12.71), indicating that the odds of SLE increased by about 9-folds for each one unit increase in protein risk score. The average AUC for 1000 BCCS reached 0.91 (95% UI: 0.88 - 0.94). In addition, the average accuracy,sensitivity, specificity and precision were all almost greater than 0.8. Specifically, the 95% UIs for accuracy,sensitivity, specificity and precision were 0.79 – 0.87, 0.73- 0.85, 0.80 - 0.91, 0.79 – 0.91, respectively (Table 2). These results indicate that the ProtRS has a good effect in diagnosing SLE, and ensure that the misdiagnosis rate and missed diagnosis rate are located within an acceptable range (Fig 2C–2H).

**Table 2:** Measurements of prediction models for 35 SLE-associated risk proteins.

Metrics	Average value	95% UI (lower)	95% UI (upper)
OR	9.197	6.807	12.705
AUC	0.911	0.882	0.937
Accuracy	0.826	0.788	0.865
Sensitivity	0.793	0.734	0.852
Specificity	0.859	0.800	0.912
Precision	0.849	0.791	0.905
F1	0.820	0.779	0.859

### Protein risk score performs more effectively in predicting SLE than polygenic risk score and clinical risk factors

**Fig 3:**
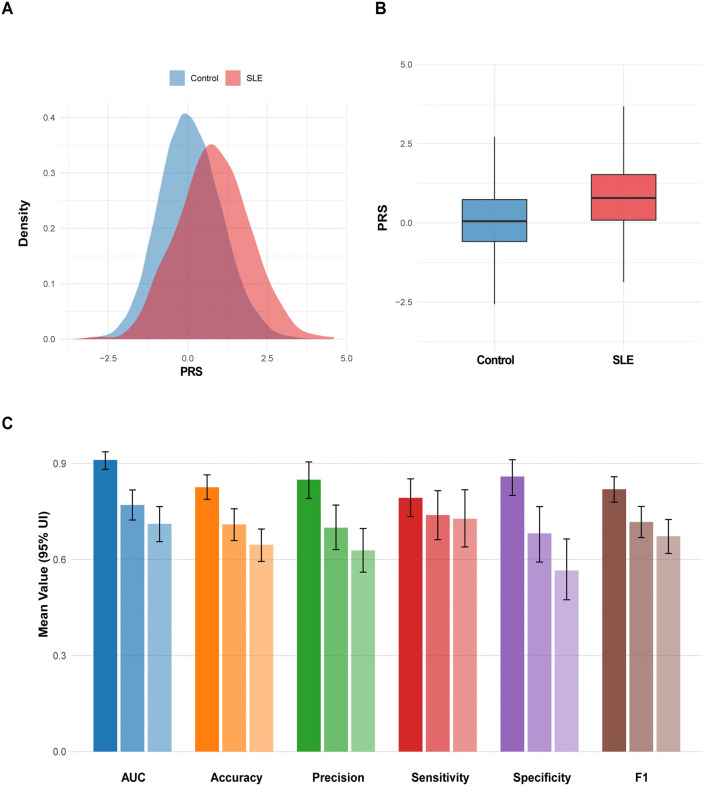
Comparison of performance of SLE prediction models based on ProtRS, PRS and established clinical risk factors. **(A)** Density plot of PRS for both SLE and control groups. **(B)** A box plot comparing PRS between two groups. **(C)** Bar plots showing the performance indicators for prediction models. For each metric, the three bars from left to right represent ProtRS-based, PRS-based, and the clinical factor based model, respectively.

The PRS, a weighted sum of multiple SNPs effect sizes, has often been used in many studies to measure the genetic risk of complex diseases. Therefore, we also explored the relationship between PRS and SLE and then assessed whether it had sufficient capacity to identify SLE patients from control individuals. It showed significant association between PRS and the disease outcome (adjusted P < 0.05) for the 1000 Logistic regression models between SLE and PRS with BCCS approach after adjusting the effect of age and gender. The OR values fluctuated from 1.49 to 2.34 with a mean value of 1.89 (95% UI:1.66 - 2.15, S3 Table), implying that SLE patients had significant higher PRS values than control individuals (Fig 3A, 3B). However, through comprehensive comparative analysis, all the indicators used to evaluate the prediction performance of the ProtRS-based model were significantly better than those of the PRS-based model (Fig 3C). For instance, the average AUC for ProtRS-based models reached 0.91 (95% UI: 0.88 - 0.94), while the PRS-based models only had an average AUC of 0.77 (95% UI: 0.72-0.82). Besides, the values of accuracy, precision, specificity, sensitivity and F1 index for ProtRS-based models almost exceeded 0.8 suggesting good classification ability. Despite of an relatively acceptable classification effect for the PRS-based models, the average specificity was 0.68 indicating a high false-positive rate of PRS in the diagnosis of SLE (S2 Fig, S3 Table). In summary, the protein risk score performs obviously better in diagnosing SLE than polygenic risk score.

In parallel with the ProtRS based model, we also constructed Logistic regression models incorporating established clinical risk factors for SLE from published literature [16, 17]. These factors included: (1) cigarette smoking status, (2) sleep duration time, (3)insomnia frequency) and (4) PM2.5 exposure levels. Comparative analysis revealed that this clinical factor model demonstrated substantially lower diagnostic performance than the protein risk score model (Fig 3C). The clinical model achieved a mean AUC of 0.71 (95% UI: 0.66-0.77), with suboptimal performance across other metrics: average accuracy = 0.65, precision = 0.63, specificity = 0.57 (S3 Fig, S4 Table). These results suggest that conventional clinical risk factors alone provide limited discriminative power for SLE diagnosis compared to protein biomarkers.

### TRIM21, SOD2, KLK3, IL15 and ADIPOQ were identified as proteins in the cost-effective optimization model for diagnosing SLE

**Fig 4:**
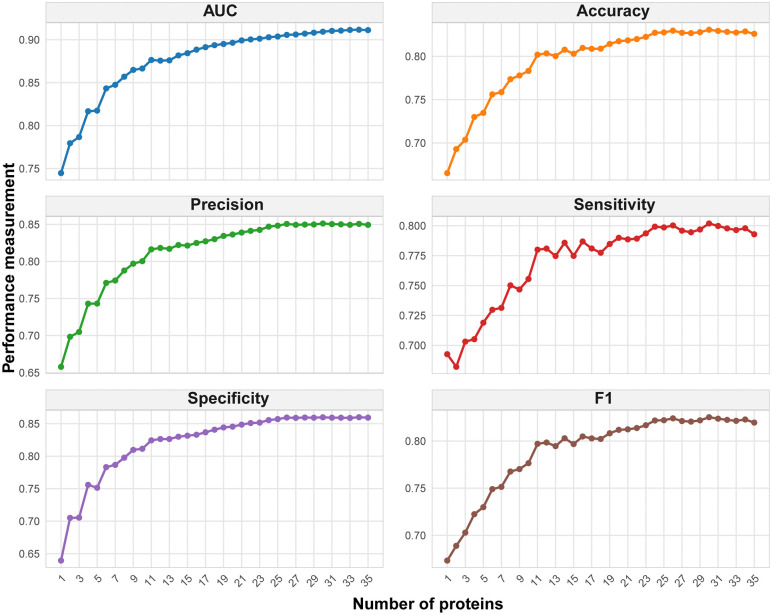
The predictive power of ProtRS-based models varies with the number of proteins. The performance of prediction models are comprehensively measured by AUC, accuracy, precision, sensitivity, specificity and F1 score.

Our prior analytical findings demonstrated that the 35-protein risk scoring model exhibited exceptional diagnostic performance for SLE, with both high balanced accuracy (AUC = 0.91) and precision (82.0%) in the validation cohort. The clinical cost of single-analyte chemiluminescence immunoassays (CLIA) for protein detection typically ranges from 80 to 300 RMB per marker, with an average of 200 RMB ($27.8) per test. Consequently, screening 35 proteins would incur a total cost of 7,000 RMB ($973), posing a significant financial burden for patients. To optimize cost-efficiency without compromising diagnostic accuracy, we identified five key proteins (TRIM21, SOD2, KLK3, IL15 and ADIPOQ) that achieved an optimal balance between economic feasibility and diagnostic performance. The resulting cost-effective diagnostic model demonstrated robust efficacy, with an AUC of 0.82 and other performance metrics were consistently close to 0.75 (Fig 4, S5 Table). Compared to the full model, this refined model reduced testing costs by approximately 86% while maintaining high diagnostic reliability, thereby substantially alleviating the economic burden on patients.

### Protein risk scores outperform both polygenic risk scores and clinical risk factors in explaining variations in SLE burden

**Fig 5:**
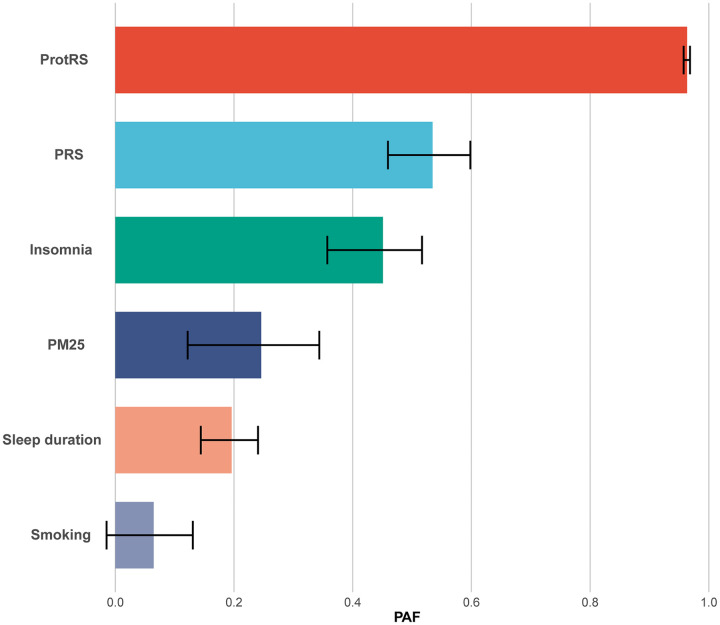
The PAF distribution which is used to represent the SLE burden caused by ProtRS, PRS, and some established clinical risk factors including insomnia, PM2.5, sleep duration and smoking.

We calculated the PAF for ProtRS, PRS and clinical risk factors including smoking status, sleep duration and quality and PM2.5 (Figs 5, S4 and S6 Table). Notably, the PAF of ProtRS far outperformed PRS and all other clinical factors, with an average PAF of 96.34% (95% UI: 95.76% - 96.84%), reconfirming the important value of plasma protein in diagnosing SLE. The influence of genetic factors on SLE that is ranked after ProtRS exceeded all clinical risk factors, and the average PAF of PRS was 53.47% (95% UI: 45.95% - 59.82%), which confirmed that SLE is a highly hereditary autoimmune disease.

Among the four risk factors, sleep quality had the greatest impact on SLE, with a mean PAF of 45.10% (95% UI: 35.71% - 51.68%), indicating that the incidence of SLE was expected to be reduced by 45.10% if the insomnia problem was completely eliminated in the target population (i.e., adjusted to a “no insomnia” state). Followed by insomnia, the average PAF of PM2.5 on SLE was 24.57% (95% UI: 12.20% - 34.38%), suggesting that if PM2.5 in the area of the target population was reduced to an ideal level, the affected individuals would theoretically reduce by 24.57%. The effect of sleep duration on SLE ranked after PM2.5, with an average PAF of 19.61% (95% UI: 14.41% - 24.06%), indicating that if the sleep duration of all individuals in the target population was adjusted to 7–8 hours, the affected individuals of SLE would theoretically be reduced by 17.54%. Smoking also had an effect on SLE, with an average PAF of 6.46% (95% UI: -1.47% - 13.05%), i.e., an expected reduction of 6.46% in individuals affected by SLE if the target population were all non-smokers.

## Discussion

In this research, we conducted a systematic evaluation of the diagnostic value of plasma protein levels for SLE, with comparative analyses against polygenic risk scores and established clinical risk factors. The results demonstrated that the ProtRS significantly outperformed both PRS and conventional risk factors in SLE diagnosis. Through 1000 BCCS iterations, ProtRS achieved remarkable performance metrics with an average AUC of 0.91 (95% UI: 0.88-0.94), sensitivity, specificity, accuracy, and precision almost exceeding 0.80. These robust findings indicate that plasma protein profiling holds substantial clinical value for SLE diagnosis, maintaining both false-positive and false-negative rates at clinically acceptable thresholds.

Through comprehensive analysis, we identified 35 high-confidence protein biomarkers (S1 Table) associated with SLE, some of which play pivotal roles in immune regulation, inflammatory responses, and the pathogenesis of autoimmune diseases. Notably, TRIM21 protein demonstrated the highest selection frequency (100%) in random sampling analyses, indicating its exceptional stability as a biomarker. TRIM21, a crucial autoantigen, has been extensively documented to associate with various autoimmune disorders, including Sjögren's syndrome, systemic lupus erythematosus, and systemic sclerosis [18]. This protein is widely expressed in multiple immune cells, such as T cells, macrophages, and dendritic cells [19]. Mechanistically, TRIM21 exhibits E3 ubiquitin ligase activity and serves as a central regulator in both innate and adaptive immunity by mediating antibody-dependent intracellular neutralization (ADIN) [20]. Interleukin-15 (IL-15), a key proinflammatory cytokine, primarily regulates the development, activation, and survival of T cells and natural killer (NK) cells [21]. Elevated IL-15 levels have been observed in SLE patients, potentially exacerbating disease progression through the activation of autoreactive T cells and the promotion of inflammatory cytokine release [22]. Given these findings, IL-15 has emerged as a promising target for immunotherapy [23]. Programmed cell death protein 1 (PD-1/PDCD1), an essential immune checkpoint molecule expressed on T cells, maintains immune tolerance by transmitting inhibitory signals. Genetic studies have revealed that polymorphisms in the PDCD1 gene are significantly associated with SLE susceptibility [24]. Functional impairment of PD-1 may lead to excessive immune activation, thereby triggering autoimmune responses. Of particular interest, kallikrein-3 (KLK3, also known as prostate-specific antigen) exhibited a high selection rate (99.1%) in this study. Although KLK3 is primarily recognized as a biomarker for prostate cancer [25], evidence suggests that single-nucleotide polymorphisms (SNPs) in its promoter region are closely linked to the development of SLE and lupus nephritis [26]. Beyond genetic associations, KLK3 is a serine protease with potential immunomodulatory functions. It cleaves insulin-like growth factor binding proteins, induces pro-inflammatory responses and inflammatory infiltrates [27, 28]. Although direct evidence in SLE is limited, its high selection frequency in our study suggests a novel role in immune dysregulation that warrants further mechanistic investigation. This finding highlights KLK3's potential as a novel biomarker for SLE risk prediction. These results not only elucidate the critical roles of these protein molecules in SLE pathogenesis but also provide a theoretical foundation for developing precision diagnostic methods and targeted therapies based on these biomarkers.

We also evaluated the impact of missing data imputation on result robustness. Besides mean imputation, we applied multiple imputation by chained equations (MICE) [29], median imputation, and k-nearest neighbors (kNN, k = 5) imputation [30], to the 35 key SLE-associated proteins. The ProtRS calculated from each alternative method showed near-perfect agreement with the original mean-imputation-based ProtRS, with Pearson correlation coefficients of 0.969 for MICE, 0.999 for median imputation, and 0.994 for kNN imputation, respectively (S5 Fig). This high consistency confirms that our findings are stable and not sensitive to the specific imputation technique employed. Accordingly, the diagnostic performance metrics (e.g., AUC) remained essentially unchanged across all imputation approaches, further supporting the validity of our protein-driven framework for SLE diagnosis (S7 Table).

While the full model by utilizing all biomarkers theoretically achieves optimal predictive performance, cost-effectiveness is required to be considered in practical clinical implementation. To bridge this gap, we systematically evaluated the trade-off between diagnostic value and economic burden by analyzing model performance with incremental biomarker inclusion (S5 Table). Our findings demonstrate that: (1) The top 10 most frequently selected biomarkers determined through 1000 BCCS contribute disproportionately to model performance, with rapid diagnostic accuracy improvement during their sequential inclusion. (2) Performance gains plateau after incorporating approximately 20 biomarkers, suggesting a limited contribution to diagnostic performance and an additional economic burden with additional markers. (3) Remarkably, even the top 5 biomarkers alone maintain clinically meaningful accuracy (AUC = 0.82, 95% UI: 0.78 - 0.86). These results support a flexible implementation framework where patients are expected to choose appropriate testing plan from tiered testing panels (5/10/20 biomarkers) based on individual economic considerations and then clinicians utilize test results to guide follow-up diagnostic and therapeutic decisions. The 5-marker panel emerges as the most cost-effective option for population-level screening. From a practical perspective, TRIM21, SOD2, KLK3, IL15, and ADIPOQ can be detected using commercial chemiluminescence immunoassay (CLIA) or ELISA kits, which are already deployed in many clinical laboratories. The typical turnaround time is 2–4 hours, allowing a same-day reporting. Thus, the 5-protein panel can be seamlessly integrated into existing diagnostic workflows without requiring specialized equipment.

The UK Biobank represents the world's largest and most comprehensive prospective cohort study, integrating large-scale, deep phenotype data with long-term follow-up, providing an unparalleled resource for investigating disease etiology and health outcomes. However, a common challenge in case-control analyses is the substantial imbalance in sample sizes between different groups, where controls may outnumber cases by tenfold or more. Such disparity can introduce bias and distort analytical results. For instance, 544 SLE cases and 48,036 controls were included in our research, in which the number of controls is 88 times larger than cases. To mitigate the impact of sample size imbalance when identifying risk biomarkers, constructing predictive models, and estimating population attributable fractions, we designed a BCCS approach with 1,000 iterations, ensuring balanced numbers of cases and controls for each analysis. This approach enhances result reliability by minimizing bias from unequal group sizes. Furthermore, to account for potential sampling variability, we performed LASSO regression with 10,000 BCCS iterations to assess biomarker stability. Only 34 proteins selected in ≥ 50% of iterations were retained, where 97.1% (33/34) of the biomarkers were consistent with results from the 1,000 BCCS replicates (S6 Fig).

Although genetic factors contribute substantially to SLE susceptibility, environmental influences, particularly sleep disturbances, also play a pivotal role in SLE pathogenesis. Our study revealed that 45.10% and 19.61% of SLE risk could be attributed to insomnia frequency and short sleep duration, respectively, ranking sleep quality as the top modifiable risk factor, which are consistent with the available epidemiological evidence. For instance, chronic low sleep duration is associated with higher SLE risk (adjusted HR 2.47, 95% CI:1.29 - 4.75) [31], less than 7 hours of sleep per night is associated with transitioning to SLE [32] and increased disease activity is associated with altered sleep architecture [33]. Sleep quality may promote the occurrence and development of SLE through the following pathways including hypothalamo-pituitary-adrenal (HPA) axis dysregulation [34], proinflammatory cytokine release [35], oxidative stress [36] and gut microbiome-mediated inflammation [37]. In this study, an SLE risk protein set with high-confidence was identified through systematic analysis, which enables early diagnosis of SLE. Insomnia intervention can be carried out for the susceptible population of SLE or early SLE patients determined through these risk markers, which would effectively reduce the burden of SLE and improve the treatment effect.

Despite the promising diagnostic performance and robustness of our ProtRS-based model, several limitations should be acknowledged. The UK Biobank cohort predominantly includes individuals of European ancestry and has an age bias toward older adults (mean age over 60 years in both control and case groups). Therefore, the generalizability of our findings to non-European populations or younger SLE patients requires further validation in independent, diverse cohorts.

## Methods

### Determination of SLE and control samples

The samples included in this study were derived from the UKB, which has collected blood, urine and saliva samples from 500,000 participants across UK since 2006, as well as comprehensive demographic, socio-economic, lifestyle and health information. Baseline plasma protein data for SLE and control individuals were used for analysis. SLE was determined based on international classification of diseases, ninth and tenth revision (ICD-9, ICD-10) with coding id of 710 and M32, and self-reported results. Individuals with other autoimmune diseases or related diseases were removed from the non-cancer self-reported population to reduce the impact of these diseases on the analysis results (S8 Table). In the end, 544 SLE patients and 48,036 control individuals with 2,923 plasma proteins were included in the study. Although the sample size of SLE cases was fixed by the UK Biobank (n = 544), our BCCS approach with 1,000 iterations balanced cases and controls at 1:1 in each iteration, ensuring statistical robustness and mitigating bias from the severe case-control imbalance (case:control = 1:88). Data centering and scaling was performed for the raw data, then the average value for one protein among all samples was used to replace the corresponding missing values.

### Clinical risk factors and polygenic risk score

Literature-reported risk factors for SLE including smoking, particulate matter (PM2.5), sleep quality were extracted from phenotype data of UKB. Regarding smoking factor, we extracted the smoking status (nonsmoking, smoking) with field id 20116. For sleep quality, sleep duration (field id: 1160) and sleeplessness or insomnia frequency (field id:1200) were selected, where sleeplessness or insomnia had three levels (never or rarely, sometimes, usually) depending the severity degree.

Standard PRS of SLE, an indicator quantifying an individual's genetic susceptibility to a certain disease or trait by integrating multiple genetic variant loci derived from GWAS, were available in UKB with the field code 26278.

### Identification of SLE-related protein markers based on LASSO regression

Given the significant disparity in sample sizes between the disease and control groups, we employed a BCCS approach to identify plasma protein biomarkers significantly associated with SLE. Specifically, in each iteration, we randomly selected a subset of controls matched in size to the SLE group, and then applied the least absolute shrinkage and selection operator (LASSO) regression to analyze the association between disease status and plasma protein levels. Proteins with non-zero regression coefficients were considered candidate biomarkers for that iteration. This process was repeated 1,000 times, and proteins selected in no less than 500 iterations (50% reproducibility) were retained as robust SLE-associated biomarkers for subsequent model construction. For each of the SLE-associated proteins, the median coefficient for the 1000 iterations was treated as the final coefficient in the following ProtRS calculation. The optimal penalization parameter λ was selected by 10-fold cross-validation using the lambda.min criterion (the λ value giving minimum mean cross-validated error). LASSO regression was performed by R package glmnet [38].

### SLE prediction model construction and performance evaluation

Similar to the marker screening process, a BCCS method was utilized to construct SLE prediction models. A subset randomly selected from the entire control cohort and SLE samples constitute data source for one iteration. The ProtRS for individual j is defined as a weighted sum of the levels for the SLE-associated proteins where β_i_ is the coefficient for protein i and Prot_ij_ refers to the value of protein i for individual j (Formula 1). The training set and testing set was randomly determined according to the ratio of 7:3. A Logistic regression model between disease state and ProtRS was constructed adjusting age and gender in the training set, and was used to predict the disease status of individuals in the test set. The performance of the SLE prediction model was comprehensively evaluated by 1000 repeated samplings with six indicators including the area under the ROC curve (AUC), accuracy, precision, sensitivity, specificity, and F1 index. The 95% uncertainty intervals (UIs) were generated for all evaluation indicators as the 2.5th and 97.5th percentiles values of 1000 random iterations.


$$\begin{array}{c} {\text{ProtRS}}_{\text{j}}=\;\sum_{\text{i}=\text{1}}^{\text{i}=\text{n}}\beta_{i}*{\text{Prot}}_{\text{ij}} \end{array}$$ (Formula 1)

### Construction of cost-effective optimization model for diagnosing SLE

The SLE-associated protein biomarkers were ranked in descending order based on their selection frequency across 1000 BCCS iterations. We then systematically evaluated the predictive performance of Logistic regression models by incrementally incorporating these biomarkers. This stepwise approach allowed us to assess how model performance varied with increasing numbers of protein features. Ultimately, we selected the most cost-effective model that achieved an optimal balance between the number of biomarkers required and diagnostic performance metrics.

### Calculation of PAF of ProtRS, PRS and clinical risk factors

Based on BCCS approach, we calculated the PAF, a commonly used indicator for disease burden studies suggesting the expected reduced proportion of affected individuals if the risk exposure reached an ideal level [39]. For discrete variables such as smoking status and insomnia, the PAF was calculated based on Formula 2, where p_i_ and RR_i_ represented the population proportion at the i’ th exposure level and the relative risk compared to the optimal exposure level, respectively. Sleep duration was divided into three categories: less than 7 hours, 7–8 hours, and more than 9 hours, and the PAF was calculated according to Formula 2 treating 7–8 hours as the optimal exposure level. The continuous features including PM2.5, PRS and ProtRS, were transformed into discrete variables by the first quartile and the third quartile and then the PAF was computed with the lowest level group as the optimal exposure. The PAF calculation is done with the PAF_calc_discrete() function in the R package graphPAF [40].


$$\begin{array}{c} \text{PAF}=\;\frac{\sum_{\text{i}}{\text{p}}_{\text{i}}{\text{(RR}}_{\text{i}}\text{- 1)}}{\sum_{\text{i}}{\text{p}}_{\text{i}}{\text{(RR}}_{\text{i}}\text{- 1)}+1} \end{array}$$ (Formula 2)

## Conclusion

A high-confidence SLE-associated risk marker set containing 35 proteins was determined. Protein risk score well distinguishes SLE from control samples and performs more effectively than polygenic risk score and clinical risk factors. A cost-optimized panel with 5 proteins (TRIM21, SOD2, KLK3, IL15, and ADIPOQ) retained high accuracy while reducing marker testing costs nearly by 87%.

## Supporting information

S1 Fig Distribution of LASSO regression coefficients for SLE-associated proteins. Note: The protein name is followed by the frequency selected in 1000 random sampling iterations. (TIF)

S2 Fig Distribution of metrics evaluating the performance for PRS-based model. It refers to the results of AUC (A), Accuracy (B), Precision (C), Sensitivity (D), Specificity (E), and F1 (F), respectively.
(TIF)

S3 Fig Distribution of metrics evaluating the performance for established clinical risk factor based model. It refers to the results of AUC (A), Accuracy (B), Precision (C), Sensitivity (D), Specificity (E), and F1 (F), respectively.
(TIF)

S4 Fig Distribution of PAF for ProtRS, PRS and established clinical risk factors. (TIF)

S5 Fig Scatter plots comparing protein risk scores (ProtRS) derived from mean imputation versus three alternative imputation methods for the 35 SLE-associated proteins. (A) MICE imputation (r = 0.969). (B) Median imputation (r = 0.999). (C) k‑nearest neighbors (kNN) imputation, k = 5 (r = 0.994). Each dot represents an individual (n = 48,580). Red line: linear regression fit; blue dashed line: y = x reference line. The high correlation coefficients indicate excellent agreement and robustness of ProtRS across different missing‑data handling approaches.
(TIF)

S6 Fig A Venn plot for comparison of effect proteins generated by 1000 subsampling iterations and 10000 subsampling iterations. (TIF)

S7 Fig Graphical abstract. A cost-pptimized protein-driven framework for early SLE diagnosis: from biomarker discovery to clinical translation. Part 1. Identification of SLE-associated plasma proteins. Part 2. Construction and performance evaluation of the SLE prediction model. Part 3. Cost-effective model optimization and PAF analysis.
(TIF)

S1 Table The detailed information for 35 SLE-associated risk proteins. (XLSX)

S2 Table The functional enrichment results for 35 SLE-associated risk proteins. (XLSX)

S3 Table Measurements of prediction models for PRS. (XLSX)

S4 Table Measurements of prediction models for established clinical risk factors. (XLSX)

S5 Table Measurements of prediction models for ProtRS-based models with protein number ranging from 1 to 35. (XLSX)

S6 Table Distribution of PAF for ProtRS, PRS and established clinical risk factors. (XLSX)

S7 Table Sensitivity analysis: diagnostic performance of ProtRS-based models using different missing data imputation methods (MICE, median, kNN) compared with mean imputation. (XLSX)

S8 Table Inclusion and exclusion criteria for diagnostic information of UKB participants in this study. (XLSX)
