# Supplementary figures and images for: A cost-optimized 5-protein panel revolutionizes systemic lupus erythematosus diagnosis
Source: PLoS Comput Biol. 2026 Jul 23;22(7):e1014513. doi: 10.1371/journal.pcbi.1014513 (PMC13395324; doi:10.1371/journal.pcbi.1014513)

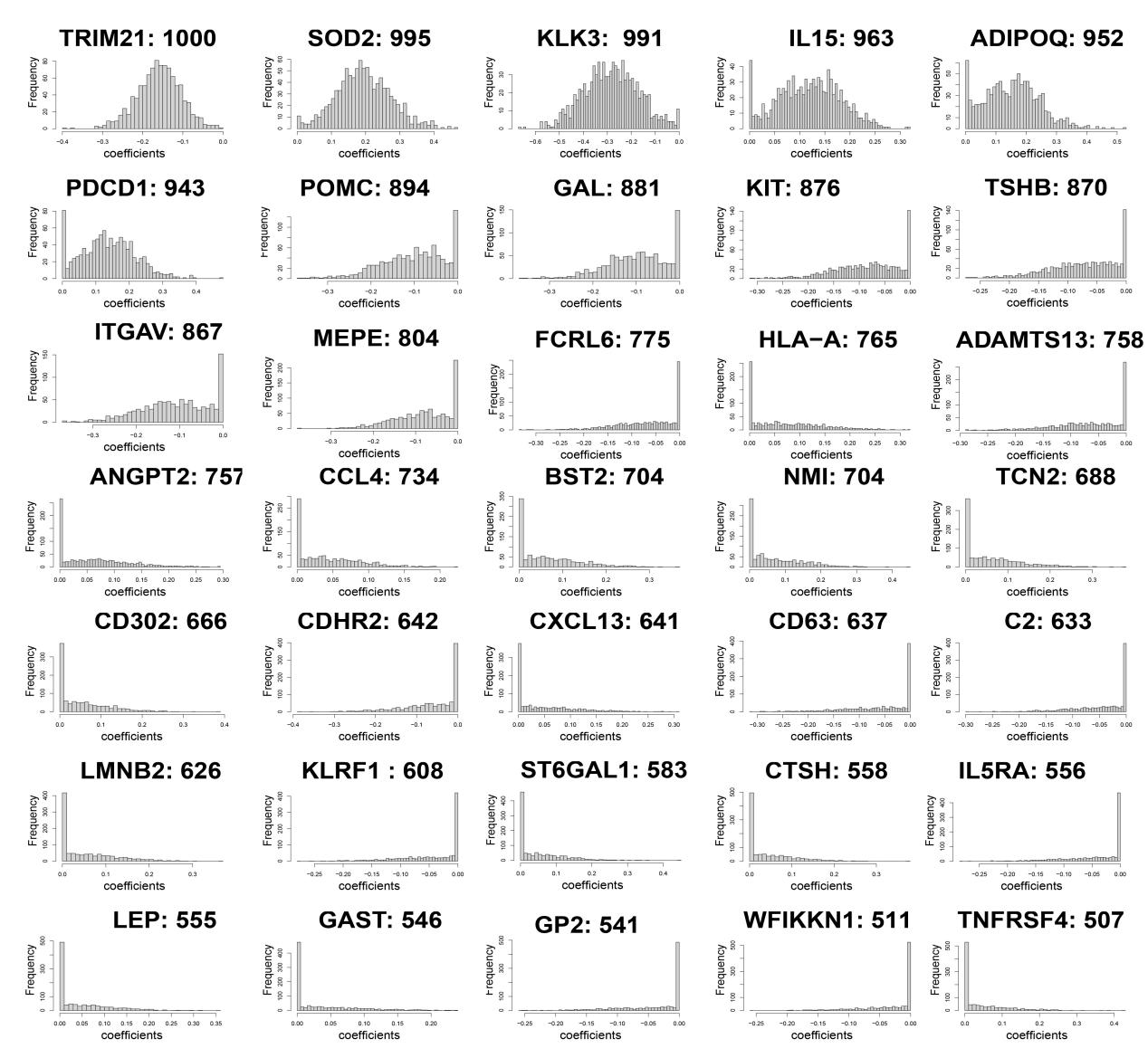

Supplement: S1 Fig — (TIF) [file pcbi.1014513.s001.tif]

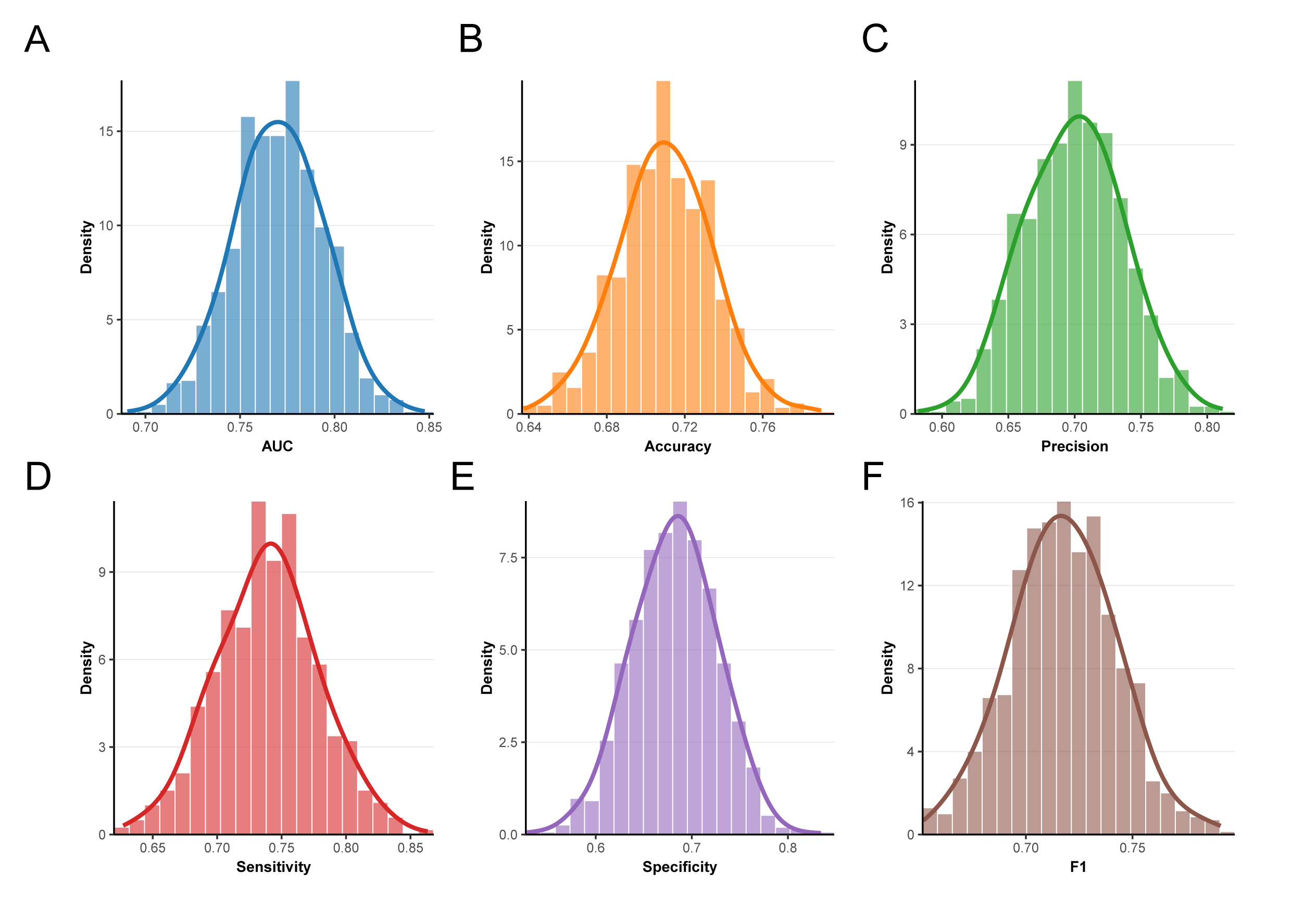

Supplement: S2 Fig — It refers to the results of AUC (A), Accuracy (B), Precision (C), Sensitivity (D), Specificity (E), and F1 (F), respectively. (TIF) [file pcbi.1014513.s002.tif]

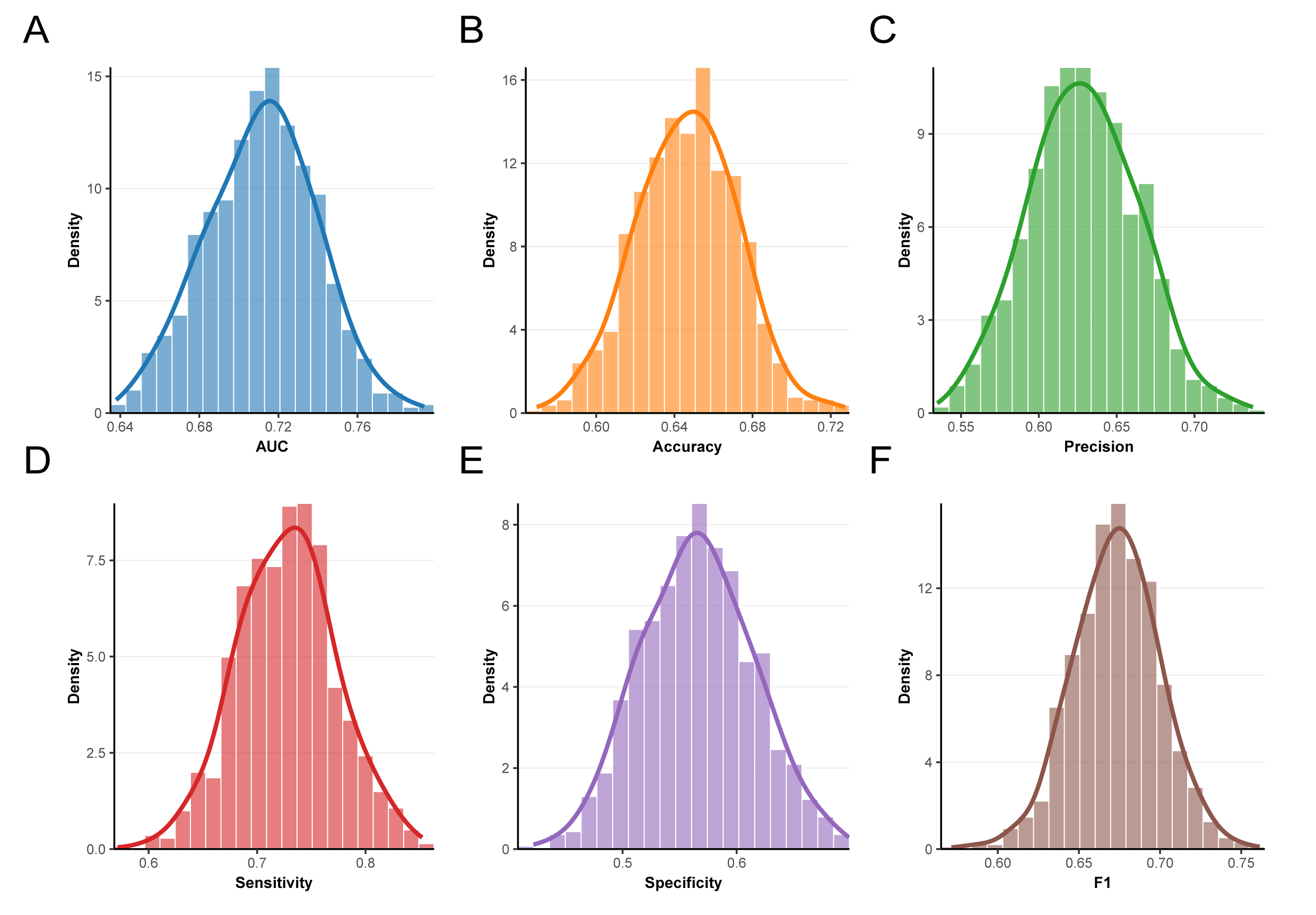

Supplement: S3 Fig — It refers to the results of AUC (A), Accuracy (B), Precision (C), Sensitivity (D), Specificity (E), and F1 (F), respectively. (TIF) [file pcbi.1014513.s003.tif]

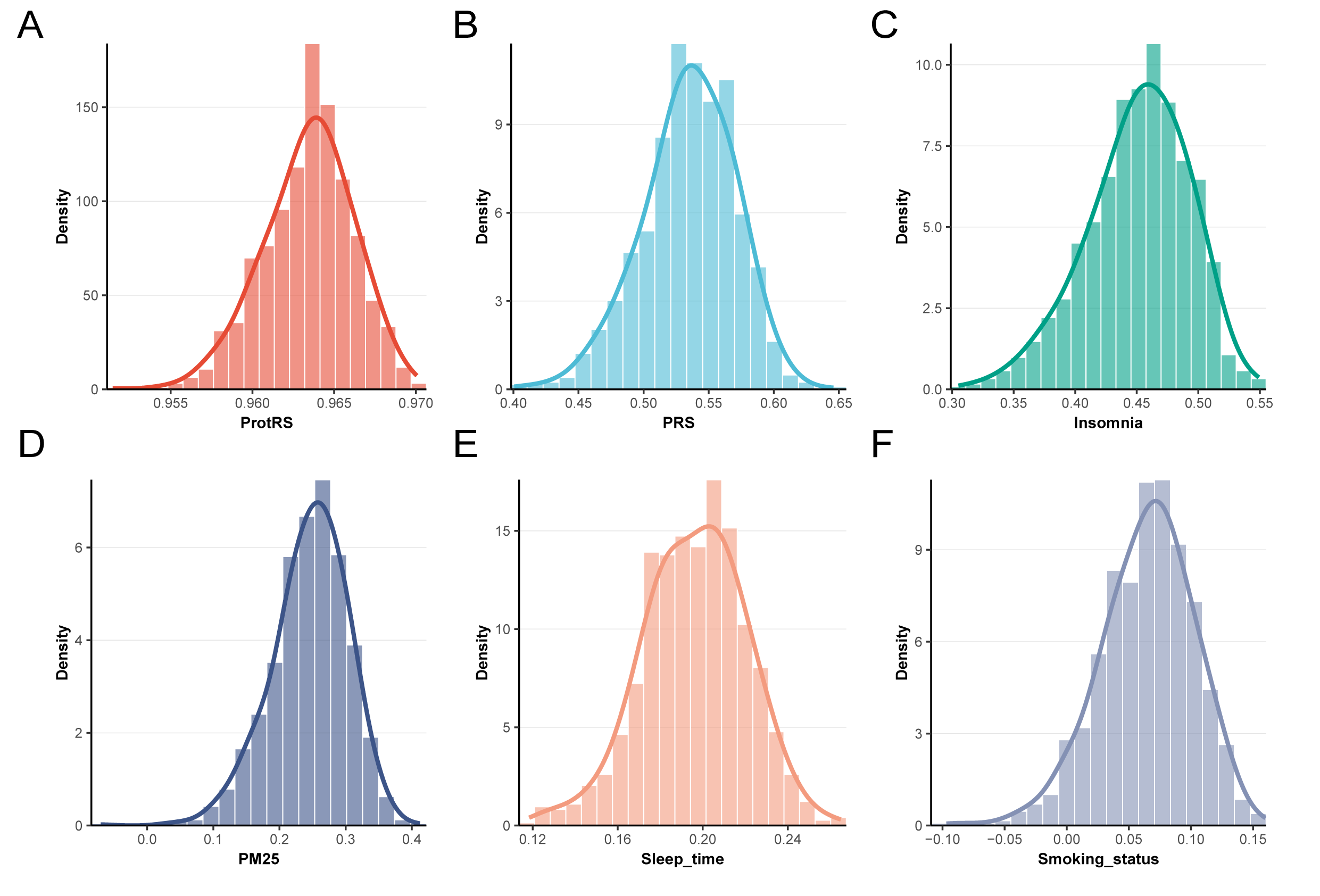

Supplement: S4 Fig — (TIF) [file pcbi.1014513.s004.tif]

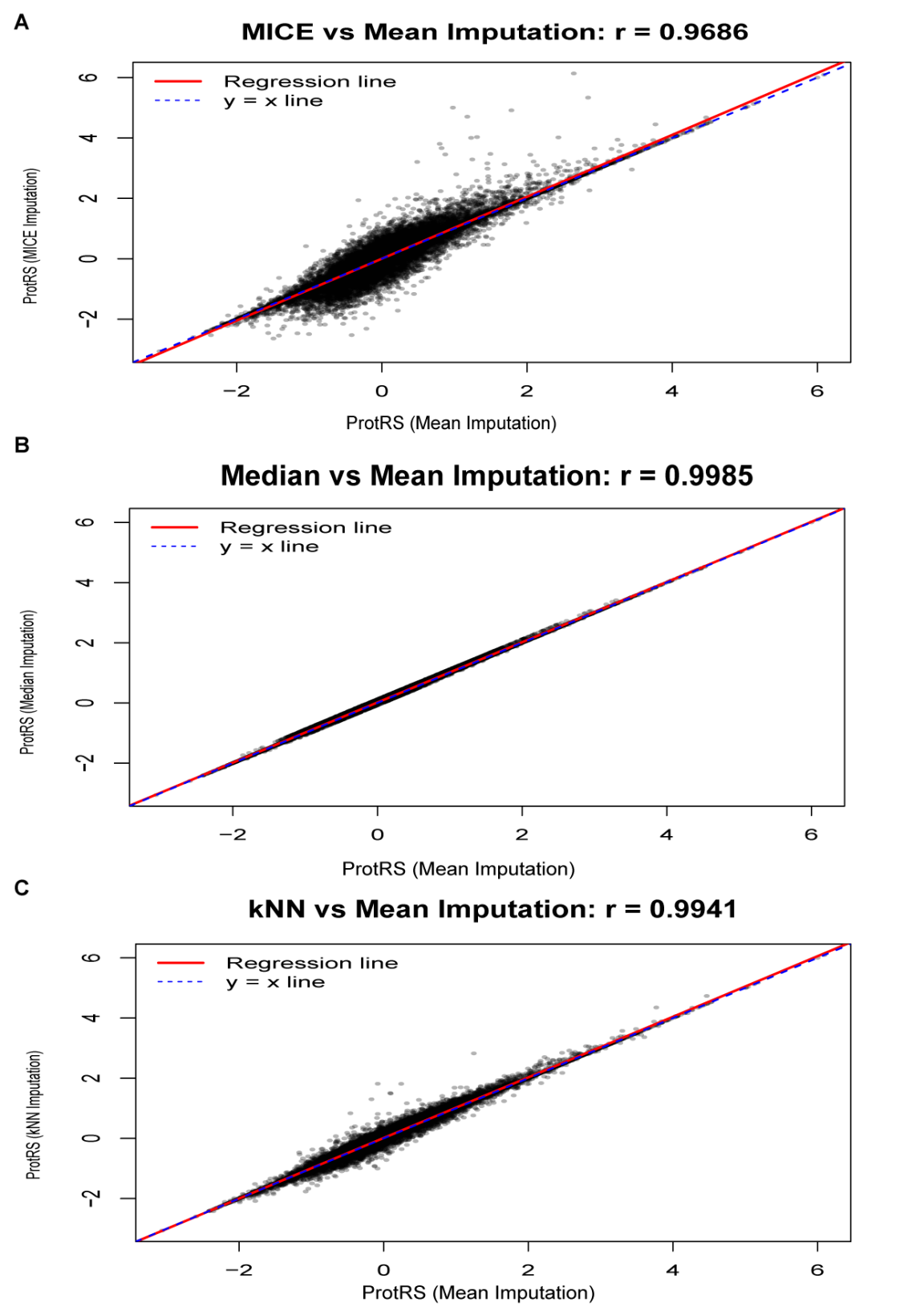

Supplement: S5 Fig — (A) MICE imputation (r = 0.969). (B) Median imputation (r = 0.999). (C) k‑nearest neighbors (kNN) imputation, k = 5 (r = 0.994). Each dot represents an individual (n = 48,580). Red line: linear regression fit; blue dashed line: y = x reference line. The high correlation coefficients indicate excellent agreement and robustness of ProtRS across different missing‑data handling approaches. (TIF) [file pcbi.1014513.s005.tif]

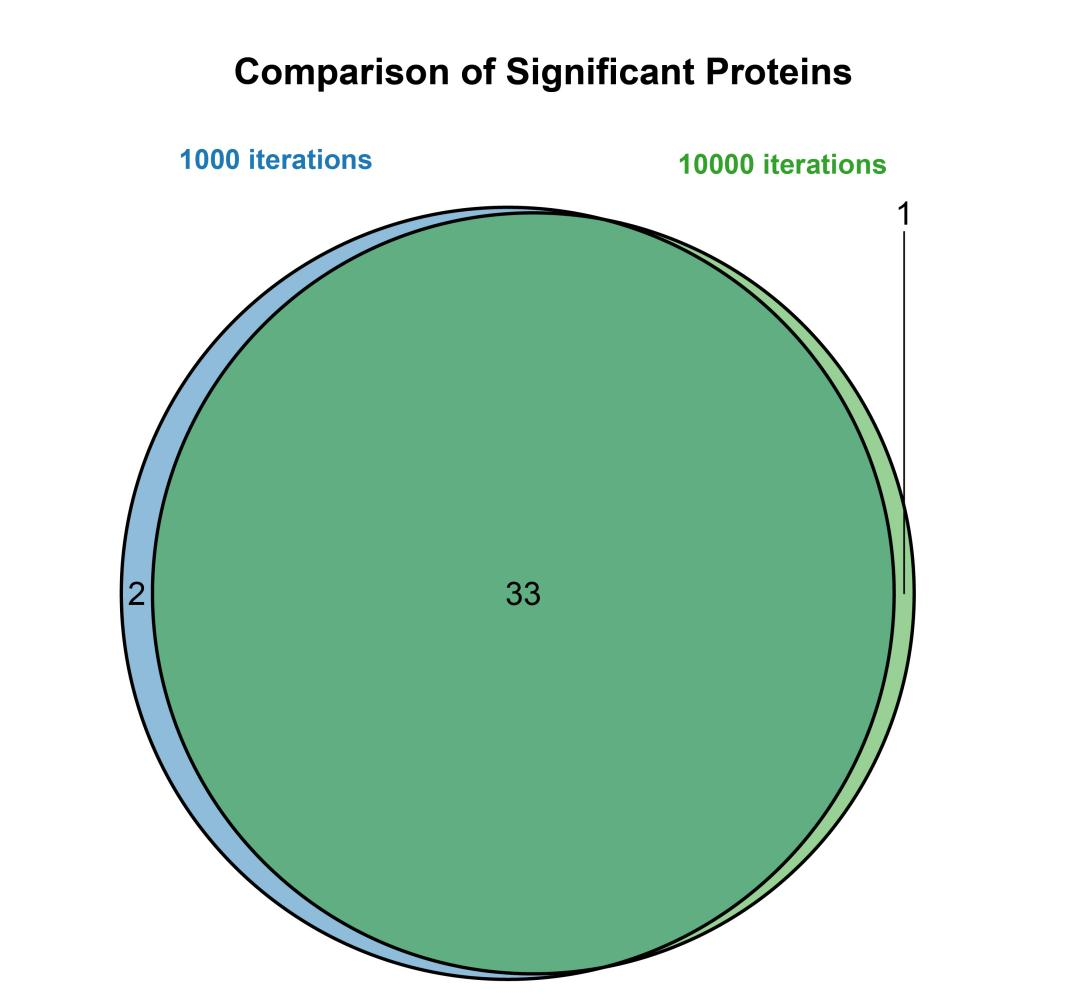

Supplement: S6 Fig — (TIF) [file pcbi.1014513.s006.tif]
